# Deformation and Failure Properties of High-Ni Lithium-Ion Battery under Axial Loads

**DOI:** 10.3390/ma14247844

**Published:** 2021-12-18

**Authors:** Genwei Wang, Shu Zhang, Meng Li, Juanjuan Wu, Bin Wang, Hui Song

**Affiliations:** 1 College of Aeronautics and Astronautics, Taiyuan University of Technology, Jinzhong 030600, China; 2 Shanxi Key Laboratory of Material Strength and Structure Impact, Taiyuan 030024, China; zhangshu0037@163.com (S.Z.); leemeng2021@163.com (M.L.); july0033@163.com (J.W.); songhui@tyut.edu.cn (H.S.); 3 College of Mechanical and Vehicle Engineering, Taiyuan University of Technology, Taiyuan 030024, China; 4 Department of Mechanical and Aerospace Engineering, Brunel University London, London UB8 3PH, UK

**Keywords:** cylindrical lithium-ion battery, axial load, failure mechanism, thermal runaway

## Abstract

To explore the failure modes of high-Ni batteries under different axial loads, quasi-static compression and dynamic impact tests were carried out. The characteristics of voltage, load, and temperature of a battery cell with different states of charge (SOCs) were investigated in quasi-static tests. The mechanical response and safety performance of lithium-ion batteries subjected to axial shock wave impact load were also investigated by using a split Hopkinson pressure bar (SHPB) system. Different failure modes of the battery were identified. Under quasi-static axial compression, the intensity of thermal runaway becomes more severe with the increase in SOC and loading speed, and the time for lithium-ion batteries to reach complete failure decreases with the increase in SOC. In comparison, under dynamic SHPB experiments, an internal short circuit occurred after impact, but no violent thermal runaway was observed.

## 1. Introduction

With the aim to reduce CO_2_ release, EV technology and EV market demand have both experienced vigorous developments to replace vehicles driven by internal combustion engines (ICEs) [1]. Among the several mainstream commercial batteries available on the market, lithium-ion batteries are favored by the EV industry due to their high energy density, good cycling performance, and lack of memory effect. However, safety accidents of lithium-ion batteries characterized by thermal runaway occur from time to time, undermining the public’s confidence in electric vehicles [2,3]. Lithium-ion batteries with lithium iron phosphate and ternary materials (mainly NCA and NCM as cathode materials) are extensively used in EVs [4] for high energy density. The probability of thermal runaway poses a serious threat of fire and explosion [5,6]. In many EV accidents, fires originate from the destruction of battery cells’ structural integrity, often caused by mechanical damage during vehicle operations, leading to violent thermal runaway [7].

In EVs, cylindrical battery cells are usually placed vertically and stacked horizontally to form modules. This unique arrangement leads to complex loading conditions. Xia et al. [8] analyzed battery fire accidents caused by foreign body collision at the bottom of EVs and established a model to study battery deformation and possible failure modes under axial loading of indenters with different shapes. Zhu et al. [9] studied axial compression experimentally and found that the force–displacement curve of the battery shows a four-stage character before short circuiting, i.e., slow rise, fast rise, slight decline, and fast rise. Finite element simulations were carried out to reveal the deformation processes, which were verified by computerized tomography (CT) scans. The researchers found that most deformation occurred at the top of the cell, with little at the bottom.

Compared with axial compression, more studies on the radial compression of cylindrical batteries are available, including the influence of SOC, SOH, loading form, and rate on battery performance. Xu et al. [10,11,12] studied the influence of SOC under quasi-static loading conditions and established a battery model showing that mechanical properties of batteries are highly dependent on SOC and weakened with a decrease in SOH. In addition, compression [13,14], three-point bending [15,16], and indentation [17] have been studied in large quantities as common loading conditions. Hao et al. [18] studied the failure process of a cylindrical lithium-ion battery under three-point bending using acoustic emission technology. Gao et al. [19,20] investigated the influence of overcharge, short-term cycle, and low-temperature charge on the mechanical integrity of battery cells subject to lateral compression. It was found that moderate overcharge has little influence on thermal runaway of the battery, while low-temperature charge reduces the crushing stress of the battery cell. Panchal et al. [21,22] studied the degradation and thermal characteristics of LiFePO_4_ batteries through experiments and established a model to analyze the attenuation of lithium iron phosphate batteries. Duan et al. [23] modeled and analyzed the heat dissipation problem of liquid cooling lithium-ion batteries. Kisters et al. [24] also conducted dynamic impact tests of pouch cells and elliptic cells and found that the failure load of elliptic cells increases with the loading speed, while the failure load of pouch cells decreases. The effect of electrolyte on the performance of an elliptic cell was also studied. It was found that the indentation of a dry cell is deeper with a higher impact velocity, but the slope of the load–displacement curve does not change much. In contrast, the depth of failure indentation in wet battery cells decreases, and the slope of the load–displacement curve increases with the increase in loading speed.

As already known, an increase in Ni content reduces the thermal safety of a battery, but few studies are available on the safety of high-nickel batteries under axial impact [25]. This paper presents an experimental study on the mechanical and safety properties of high-nickel batteries under both static and dynamic axial load experiments. CT scan was used to detect the internal damage of the batteries after the tests. This study is a step toward understanding the safety behavior of lithium-ion batteries under different axial loads.

## 2. Experimental Section

### 2.1. Information of the Cell

**Figure 1:**
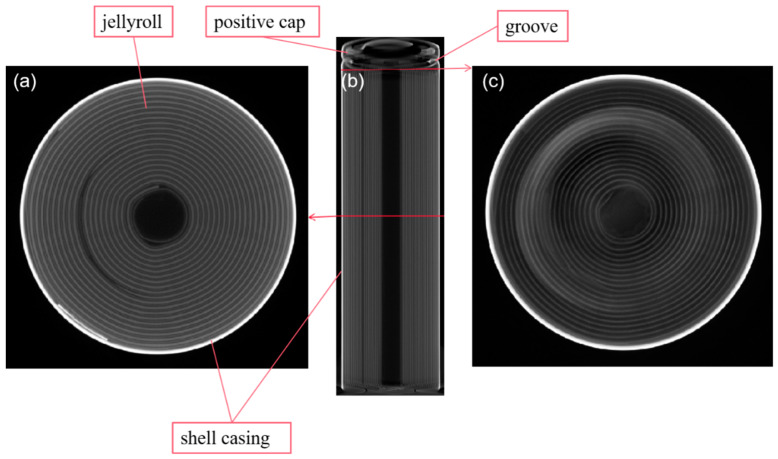
CT inspection of the battery studied: (**a**) cross-section of the midsection; (**b**) side view; (**c**) cross-section of the top section.

A commercial model of cylindrical high-nickel lithium-ion cells with LiNi_0.8_Co_0.1_Mn_0.1_O_2_ as cathode material was used in this study. The battery is 65 mm in length and 18 mm in diameter with a nominal energy capacity of 3050 mAh and a nominal voltage of 3.6 V. The jellyroll is shown in Figure 1, which includes an anode, a cathode, a collector, and a separator comprising the internal structure of the cell.

### 2.2. Experimental Methods

Before the tests, all batteries were charged to a fixed SOC. During charging and discharging, the C-rate is a commonly used term to express the charge and discharge rates. The C-rate is equal to the charge or discharge current divided by the rated capacity. At this stage, all batteries were charged in the CC-CV mode [26]. In this process, batteries were first charged at 0.2 C to a rated voltage of 4.2 V, charged at a constant voltage of 4.2 V until the current was less than 0.02 C, then discharged at a constant current of 0.2 C to the cut-off voltage. After standing, they were charged again at a constant rate of 0.2 C to different SOCs.

**Figure 2:**
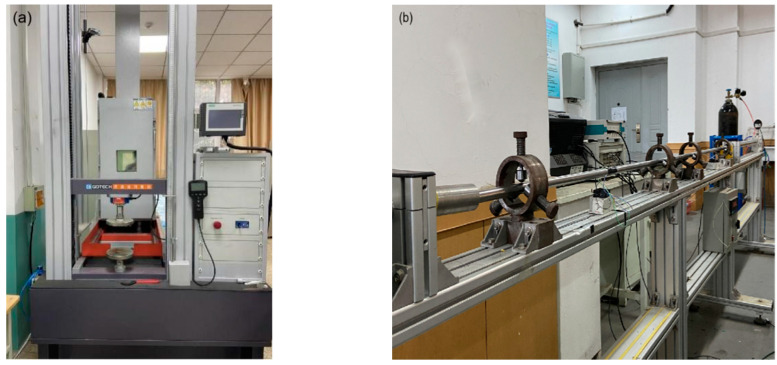
(**a**) Gotech testing machine; (**b**) SHPB system set-up.

A quasi-static test was then carried out using a Gotech screw-driven testing machine (Gotech Testing Machines Inc., Taiwan, China) with a loading capacity of up to 30 kN, as shown in Figure 2a. During the test, voltage changes of the battery were recorded by a digital oscilloscope. The battery was connected with a digital oscilloscope to monitor the voltage change, then the battery was placed on the testing machine and loaded at a specified loading rate. Temperature changes of the battery were recorded by an infrared thermal imager (Yoseen X640A600MF25, Wuhan Yoseen Infrared Co., Ltd., Wuhan, China).

In the dynamic axial impact test, a 19 mm diameter SHPB system was used, and a high-speed camera (I-Speed 716, iX Cameras Ltd., Rochford, England) was used to record the deformation process with a sampling rate of 75 kHz, as illustrated in Figure 2b. The battery was placed axially between the incident bar and a fixed plate. A laser velocimeter was used to measure the speed of the striking bullet, which hit the far end of the incident bar to apply a compressive stress wave on the battery. Each test was repeated three times, and the mean value was used to reduce experimental uncertainty.

## 3. Results

### 3.1. Quasi-Static Experiment

Batteries with 0%, 20%, 40%, 60%, and 80% SOCs were tested. The crosshead displacement loading was set at a constant speed of 2 mm/min. In order to determine the influence of loading speed on thermal runaway, additional compression tests at 8 mm/min were also carried out on cells with 40% and 60% SOCs.

**Figure 3:**
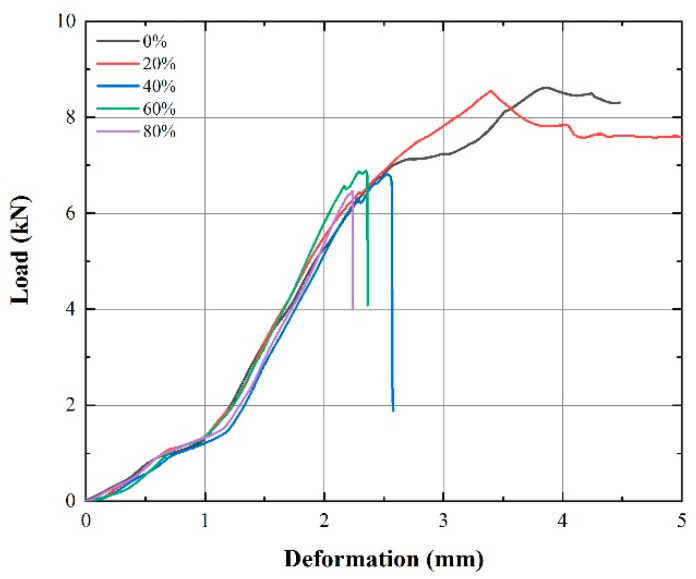
Load–deformation curves of batteries with 0%, 20%, 40%, 60%, and 80% SOCs under axial compression at 2 mm/min.

Figure 3 shows the load–deformation curves of lithium-ion batteries with different SOCs. With increasing deformation, loads on the batteries with different SOCs show a virtually identical upward trend in the early stage. When deformation exceeded 2 mm, batteries with 40% SOC and above soon reached the peak load and failed, indicated by a sudden loss (dive) in voltage. Batteries with 0% and 20% SOCs deformed further with a plateau load with further deformation after reaching the peak load.

**Figure 4:**
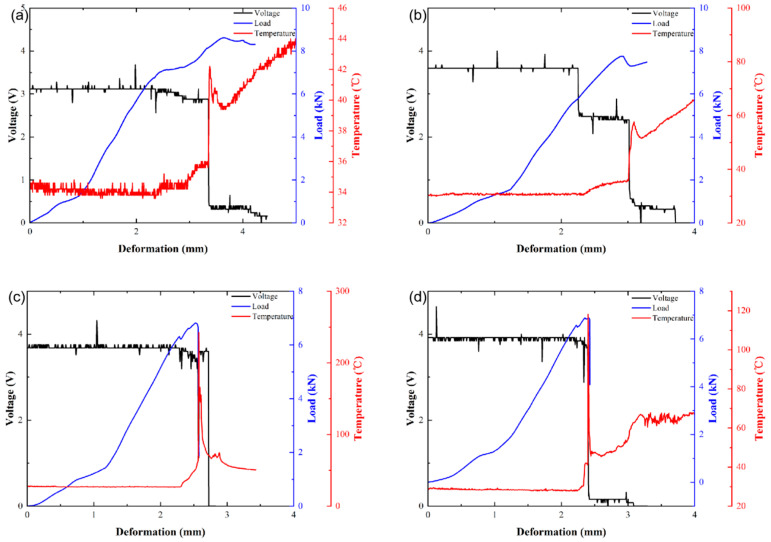

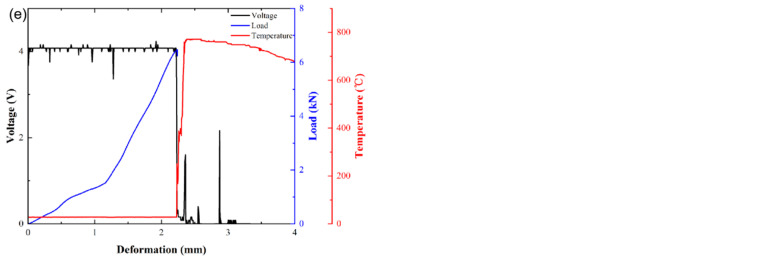
Voltage, load, and temperature–deformation curves of batteries with different SOCs: (**a**) 0%; (**b**) 20%; (**c**) 40%; (**d**) 60%; (**e**) 80%.

Temperature and voltage changes in the batteries were collected with respect to the deformation. Figure 4a–e shows the voltage, load, and temperature of batteries with 0%, 20%, 40%, 60%, and 80% SOCs. It can be seen that all batteries with a higher SOC experienced thermal runaway.

**Figure 5:**
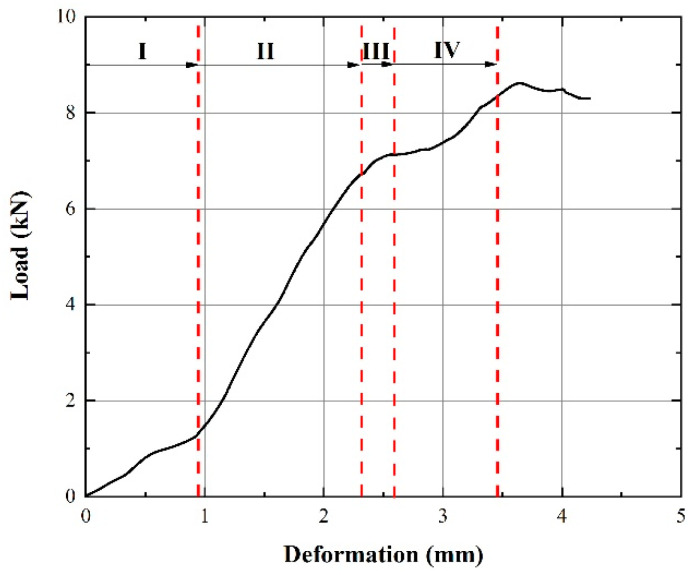
Stages of the load–deformation curve of a battery with 0% SOC under axial compression.

The load–deformation curve of a 0% SOC battery is further analyzed in Figure 5. The compression process can be divided into four stages. In Stage I, the groove of the positive cap at the battery top is flattened with the cap just in contact with the jellyroll. In Stage II, the jellyroll is pushed and compressed by the positive cap and shows increasing resistance. In Stage III, the jellyroll starts to bend and buckle in the lateral direction, resulting in the initiation of internal short circuiting. In Stage IV, the local area of the jellyroll deforms excessively, and internal short circuiting becomes more severe. This is similar to the experimental results of Zhu et al. [9], but the voltage change is significantly different, which is caused by the difference in the inner structures of the battery and the properties of different electrode materials.

As shown in Figure 4c,d, the temperature of batteries with 40% and 60% SOCs begins to rise, and the voltage drop starts in Stage III. At the end of Stage III, the temperature rises sharply, and the battery reaches the failure state. In comparison, batteries with 80% SOC show a sudden temperature rise and a complete voltage drop at the end of Stage II (Figure 4e), and the maximum temperature reaches nearly 800 °C. It can clearly be seen that the severity of thermal runaway increases with the SOC.

**Figure 6:**
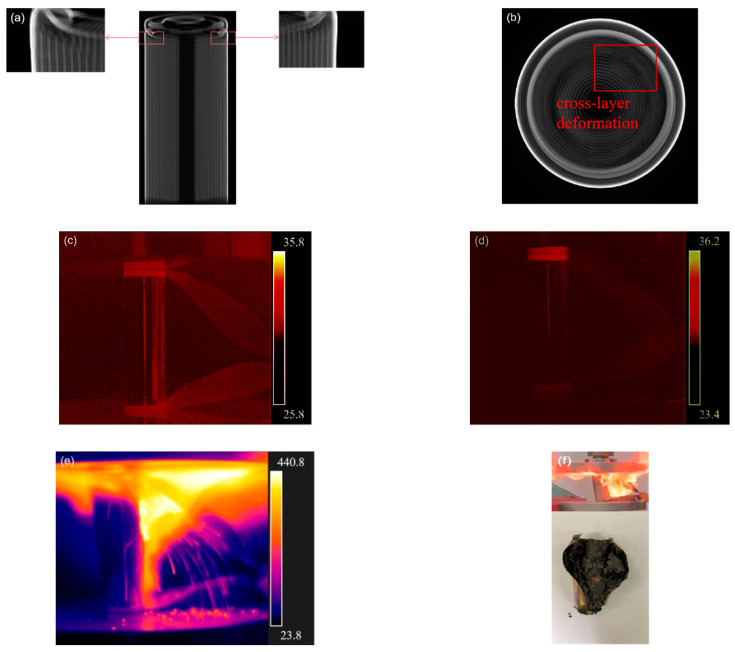
At the end of Stage II: (**a**) CT inspections of side view; (**b**) CT scan cross-section close to the positive cap; (**c**) temperature nephogram of the battery with 0% SOC; (**d**) temperature nephogram of the battery with 40% SOC; (**e**) temperature nephogram of the battery with 80% SOC; (**f**) flame in 80% SOC battery during test and the aftermath.

CT inspections at the end of Stage II of the longitudinal section in Figure 6a show that the jellyroll was squeezed by the positive cap and began to deform. With part of the jellyroll buckled, gaps appeared in the negative current collector, as shown in Figure 6b, and cross-layer deformation occurred in the jellyroll near the top of the battery, yielding in direct contact with the positive and negative poles. Figure 6c–e shows the Joule heat generated around the contact area with the increased potential of thermal runaway. The short-circuit current in the damaged area increases with the battery’s SOC, resulting in an increase in Joule heat and a higher temperature. Therefore, batteries with excessively high SOCs experience severe thermal runaway, such as those of batteries with 80% SOC, as shown in Figure 6e. In Figure 6f, it can be seen that the battery erupted into flames near the positive electrode.

**Figure 7:**
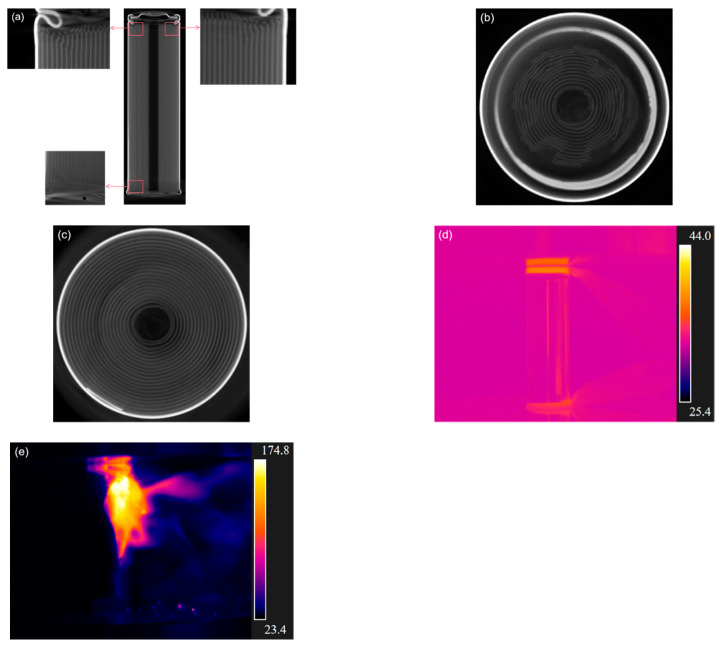
At the end of Stage III: (**a**) CT scan of side view at the end of Stage III; (**b**) CT scan of the cross-section near the positive cap; (**c**) CT scan of the cross-section near the negative pole; (**d**) temperature nephogram of the battery with 0% SOC; (**e**) temperature nephogram of the battery with 40% SOC.

Figure 7 shows the deformation and temperature of the battery loaded at the end of Stage III. In Figure 7a, the jellyroll was further compressed, resulting in deformation at both the top and bottom parts of the battery. The cross-layer deformation of the jellyroll near the top of the battery is shown in Figure 7b. The increased contact area between the positive and negative poles led to the electric failure of the battery. At this stage, voltage drops occurred in batteries with 40% and 60% SOCs, respectively. As shown in Figure 7c, the jellyroll near the negative pole remained almost intact, with deformation largely limited to the casing of the battery.

Temperature was seen higher near the positive cap of the 0% SOC battery (Figure 7d) than that at the end of Stage II (Figure 6c). As shown in Figure 7e, the flame bursts near the positive cap of the battery with 60% SOC, and temperature rises abruptly.

**Figure 8:**
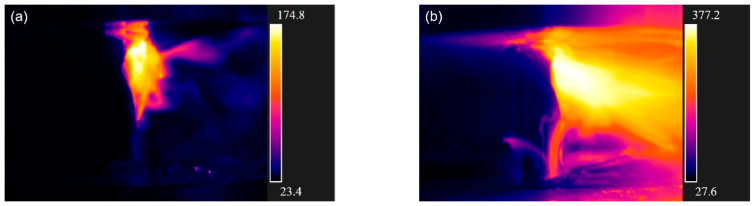

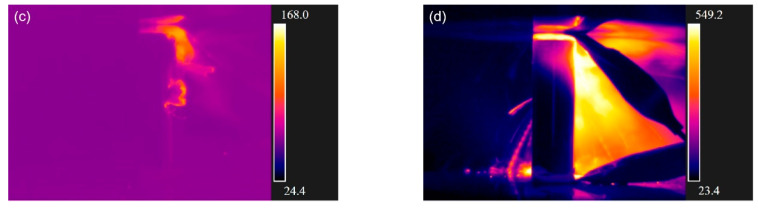
Temperature nephogram under thermal runaway: (**a**) battery with 40% SOC at 2 mm/min; (**b**) battery with 40% SOC at 8 mm/min; (**c**) battery with 60% SOC at 2 mm/min; (**d**) battery with 60% SOC at 8 mm/min.

Further tests were carried out with a higher loading rate at 8 mm/min on batteries with 40% and 60% SOCs. A comparison of test outcomes is given in Figure 8 and Table 1.

As shown in Figure 8, the intensity of thermal runaway is more serious under the speed of 8 mm/min. It can be seen that thermal runaway occurred at a higher temperature in batteries with 40% SOC, and it occurred to a larger extent but with no flame. The battery with 60% SOC did not catch fire at the loading rate of 2 mm/min, but when the loading rate was 8 mm/min, it burned and burst into flames. Table 1 shows that the peak temperature increases significantly under the loading speed of 8 mm/min. It is noticed that batteries with 60% SOC burst into flames under 8 mm/min, but not 2 mm/min. This is because the increase in the short-circuit area is slower in the latter, resulting in a slower accumulation of Joule heat. It can be concluded that the intensity of thermal runaway increases with the increase in the loading speed.

### 3.2. Axial Impact Experiment (SHPB)

Compared with the quasi-static axial compression tests, a 19 mm diameter SHPB device was used to investigate the dynamic response of the batteries. Three striking velocities were selected for the bullet at 9.1 m/s, 11.6 m/s, and 13.1 m/s. Lithium-ion batteries with 20%, 40%, 60%, and 80% SOCs were tested to explore the mechanical response and failure modes.

Pulse signals in the pressure bar were recorded by a strain gauge on the surface of the incident bar, which was used to calculate the dynamic force impacted on the battery using the following formula:

(1) $$F=EA\left(\varepsilon_{i}+\varepsilon_{r}\right)$$

where $\varepsilon_{i}$ and $\varepsilon_{r}$ are the incident and reflected strains, respectively. *E* is the elastic modulus of the pressure bar, and *A* is the cross-sectional area of the pressure bar. Again, tests were repeated three or more times to obtain mean values and error estimates on the outcome.

**Figure 9:**
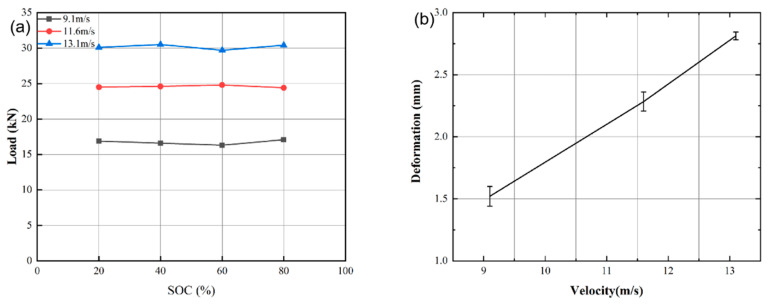
(**a**) Mean peak impact force on batteries with different SOCs at three striking velocities; (**b**) mean residual deformation with error bar of batteries with 40% SOC vs. impact velocity.

Figure 9a shows the mean peak impact loads of batteries with 20%, 40%, 60%, and 80% SOCs at different striking velocities. The measured loads are seen to have little correlation with the SOC, but both the load and the deformation increase clearly with the impact velocity. Figure 9b shows the mean values and error bars of the residual deformation of the recovered batteries with 40% SOC with respect to the impact velocity. The mean residual deformation is seen to increase approximately linearly with the velocity.

**Figure 10:**
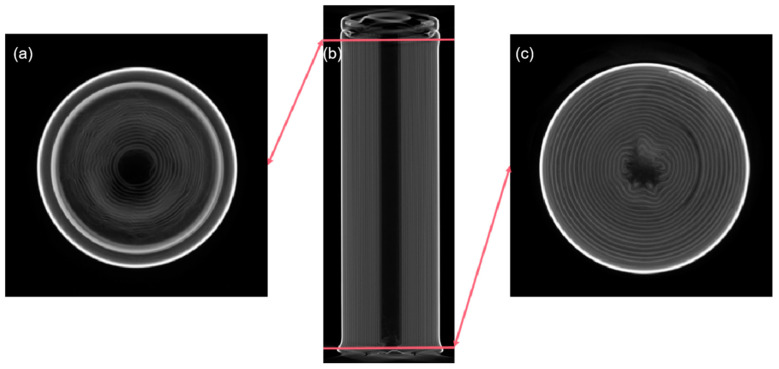
CT inspections after axial impact under 11.6 m/s impact: (**a**) cross-section of the top section; (**b**) side view; (**c**) cross-section of the bottom section.

Figure 10 shows the CT scans of a tested lithium-ion battery impacted at 11.6 m/s. It can be seen that deformation occurred at both the positive and negative ends of the battery (Figure 10a,c). The main part of the battery in the middle did not show signs of deformation (Figure 10b). The CT scans show that the jellyroll near the positive cap had deformation, but there was less cross-layer deformation than that in quasi-static tests (Figure 7b,c). The central region of the jellyroll at the negative end was also wrinkled with cross-layer deformation. This may yield internal short circuiting of the battery. There was no severe thermal runaway, and no obvious temperature rise was observed in the dynamic tests, which is rather different from the outcome of the quasi-static tests, even though the overall deformation is comparable in the two loading scenarios.

### 3.3. Comparison between Dynamic and Quasi-Static Loading

Based on the experimental outcome, different loading mechanisms can affect the failure forms of batteries. The response of batteries under dynamic and quasi-static loading differs in the following aspects:

(a) Under quasi-static load, the deformation of the battery is mainly concentrated in the positive cap area of the battery with cross-layer deformation in the jellyroll, and little deformation exists at the negative end. Under dynamic impact load, deformation occurs at both the positive and negative electrode ends, but the jellyroll near the positive cap suffers much less cross-layer deformation, and the central region of the jellyroll at the bottom end is wrinkled.
(b) Under quasi-static load, the carrying capacity of the battery is in the range of 6–8 kN, which varies slightly with the SOC. In contrast, the peak transient load capacity of the battery exceeds 15 kN under dynamic impact load.
(c) The failure modes of the battery under the static and dynamic loading are different, even with similar levels of deformation. Thermal runaway occurs under quasi-static loading and is related to the level of SOC and the loading velocity. Under dynamic loading, however, only minor internal short circuiting occurs with no thermal runaway.

## 4. Discussion

Under excessive mechanical loading, the occurrence of thermal runaway should be attributed to the various side reactions. Specifically, when abnormal heat builds up inside the battery, a chain of chemical reactions takes place. At first, mechanical abuse causes internal structural damage and internal short circuits, which raise the temperature. When the temperature is beyond 90 °C, lithium salt and SEI film start to decompose. The side reactions caused by abnormal heat accumulation continue to release more heat and form a heat-temperature cycle leading to a fire burst [27,28].

The experimental results show that, under quasi-static axial compression load, the voltage of the battery drops slightly at first in the axial compression process until a complete sudden short-circuit. When the voltage drops for the first time, the temperature at the positive and negative electrode sides begins to rise slightly. Meanwhile, an internal short circuit occurs at the positive and negative poles of the battery. When the battery voltage drops completely, the temperature of the battery rises sharply.

For quasi-static loading, the intensity of battery thermal runaway is different with respect to the level of SOCs. This is because the short-circuit current in the damaged area increases with the battery’s SOC, resulting in an increase in Joule heat. Therefore, batteries with excessively high SOCs will experience severe thermal runaway, such as batteries with 80% SOC. When the electric capacity is lower at, for instance, 40% or 60% SOCs, at early loading stages, only smaller areas of the battery are damaged, and the short-circuit current is not big enough to generate sufficient Joule heat to reach the temperature that causes thermal runaway. However, when the battery is further deformed with bigger damaged areas, more Joule heat is generated, which causes thermal runaway.

The intensity of thermal runaway increases with the loading speed. The faster the loading speed, the larger the short-circuit area per unit time, the more Joule heat accumulated, and the more likely it is to have thermal runaway, as shown by the experimental outcome of batteries with 60% SOC.

In comparison, the experimental results show that under dynamic loading, battery failure modes can be quite different from those under quasi-static loading. Under dynamic impact, the internal damage area of the battery is smaller, resulting in less Joule heat generated by internal short circuits, thus no thermal runaway.

Compared with LiCoO2 [9] and ordinary NCM [29] batteries, it is found that the failure displacement of high-nickel batteries is smaller when axially loaded and easier to burst into flames. The safety of high-nickel batteries still needs to be further improved.

## 5. Conclusions

In this paper, both quasi-static and dynamic axial compression tests were carried out on Hi-Ni lithium-ion batteries with different SOCs. Battery voltages, loading forces, and temperatures in quasi-static axial compression were recorded, and battery failures and thermal runaway intensities were analyzed. In SHPB impact tests, residual deformation and peak loads were recorded. The failure modes of batteries under the two loading forms were compared. The results show that the effect of mechanical loads on the safety performance of cylindrical 18,650 lithium-ion cells depends significantly on the loading form (i.e., either quasi-static loads or dynamic impact). Under quasi-static axial compression, severe thermal runaway occurs in batteries with high SOCs, but not under dynamic impact load for comparable overall deformations. In addition, under quasi-static axial compression, the intensity of thermal runaway becomes more severe with the increase in SOC and loading speed.

The results shed light on the failure mechanism of lithium-ion batteries under axial load and guide the safety design of the battery and safety arrangement of battery packs.

## Figures and Tables

**Table 1:** Experimental results at different loading speeds.

SOC	Loading Rate (mm/min)	Thermal Runaway	Fire	Peak Temperature (°C)
40%	2	Yes	No	242
40%	2	Yes	No	174
40%	2	Yes	No	190
40%	8	Yes	No	330
40%	8	Yes	No	443
40%	8	Yes	No	301
60%	2	Yes	No	182
60%	2	Yes	No	168
60%	2	Yes	No	---
60%	8	Yes	Yes	549
60%	8	Yes	Yes	649
60%	8	Yes	Yes	672

## Data Availability

Not applicable.

## Nomenclature

BMS	Battery management system
BTMS	Battery thermal management system
CC	Constant current
CT	Computerized tomography
CV	Constant voltage
EV	Electric vehicle
ICE	Internal combustion engine
LIB	Lithium-ion battery
NCA	Lithium nickel-cobalt-alumina oxide
NCM	Lithium nickel-cobalt-manganese oxide
SHPB	Split Hopkinson pressure bar
SOC	State of charge
SOH	State of health
